# Neonatal Readmissions to a Level 4 Hospital

**DOI:** 10.1111/jpc.16053

**Published:** 2022-06-20

**Authors:** Olivia Jarrett, Wafa Kulsoom Khan, Anthony Liu, Habib Bhurawala

**Affiliations:** ^1^ Department of Paediatrics Nepean Hospital, Nepean Blue Mountains Local Health District Sydney New South Wales; ^2^ Discipline of Paediatrics The University of Sydney Nepean Clinical School, Faculty of Medicine and Health, The University of Sydney Sydney New South Wales

Dear Editor,

## NEONATAL READMISSIONS TO A LEVEL 4 HOSPITAL

Further to our published article,^1^ we write in response to Dr Gribble,^2^ who questioned whether weight loss during the birth admission was greater in babies whose mothers received intravenous fluid during labour and whether these babies were more likely to need readmission for weight loss. Dr Gribble suggested a delay in weighing until 12–24 h after birth to allow time for babies to experience diuresis and correct their fluid status.

Out of the 129 babies who required readmission, only 23 of these babies were subsequently weighed before discharge following birth. Although the numbers are small, we found that babies whose mothers received intravenous fluids during labour had less average weight loss than those whose mothers did not receive intravenous fluids (6% vs. 8%, respectively, *P* = 0.4). However, the relationship between maternal intravenous fluids during labour and weight loss in babies is not established^3^, ^4^, ^5^, ^6^ and is an area that requires ongoing research.

There is no widely accepted protocol for the timing of newborn weights whilst in hospital following the birth or after discharge. At our hospital, the average length of stay following a normal vaginal delivery and an uncomplicated caesarean section is 2.1 days. The current practice is to weigh a baby at birth only unless otherwise clinically indicated. This means that only the birthweight can be considered a baseline weight when assessing weight change, despite a suggestion that weight after 24 h should be used instead.^3^ Further research into the relationship between maternal intravenous fluids during labour and baby weight loss is required. It may influence hospital policies on the timing of baby weights and weight loss assessment.
